# Effect of ultraviolet C radiation on biological samples

**DOI:** 10.3325/cmj.2013.54.263

**Published:** 2013-06

**Authors:** Branka Gršković, Dario Zrnec, Maja Popović, Maja Jelena Petek, Dragan Primorac, Gordan Mršić

**Affiliations:** 1 Forensic Science Centre “Ivan Vučetić”, General Police Directorate, Ministry of Interior, Zagreb, Croatia; 2 University Center for Forensic Sciences, University of Split, Split, Croatia; 3 Faculty of Food Technology and Biotechnology, University of Zagreb, Zagreb, Croatia; 4 Department of Biology, Faculty of Veterinary Medicine, University of Zagreb, Zagreb, Croatia; 5 University of Split School of Medicine, Split, Croatia; 6 University of Osijek School of Medicine, Osijek, Croatia; 7 Eberly College of Science, Penn State University, University Park, PA, USA; 8 University of New Haven, New Haven, CT, USA; 9 Genos Ltd, Zagreb, Croatia

## Abstract

**Aim:**

To examine the influence of ultraviolet C (UVC) radiation on blood, saliva, semen, and naked DNA samples for preventing DNA cross-contamination on working surfaces in laboratories.

**Methods:**

Blood, saliva, semen, and DNA isolated from buccal swab samples were obtained from a single male donor and applied to the laboratory working surfaces. UVC radiation was applied to these diluted and undiluted samples with or without previous decontamination of the working surfaces with 10% sodium hypochlorite and 20% ethanol. Genomic DNA was extracted using Chelex. After quantification, DNA was amplified using the AmpFlSTR® NGM™ PCR Amplification Kit. We tested and statistically analyzed DNA concentration, UVC dose, sample volume, radiation time, the number of correctly detected alleles on genetic loci, and the number of correctly detected alleles in four groups in which 16 loci were divided.

**Results:**

When working surfaces were not decontaminated and were treated only with UVC radiation in the laboratory, the genetic profile for naked DNA could not be obtained after 2 minutes of UVC radiation and for saliva after 54 hours. For blood and semen, a partial genetic profile was obtained even after 250 hours of UVC radiation in the laminar. When working surfaces were decontaminated with 10% sodium hypochlorite and 20% ethanol, genetic profile could not be obtained for naked DNA after 2 minutes, for saliva after 4 hours, for blood after 16 hours, and for semen after 8 hours of UVC radiation in the laboratory.

**Conclusion:**

It is recommended to carefully and thoroughly clean working surfaces with 10% sodium hypochlorite and 20% ethanol followed by minimal 16-hour UVC exposure (dose approximately 4380 mJ/cm^2^) for complete and successful decontamination.

Advances in forensic genetics have enabled DNA profile identification from minute DNA amounts (1) and degraded DNA samples (2). Due to an increasing number of cases, contamination is becoming one of the major problems in forensic casework analysis. Contamination of forensic evidence with foreign DNA can result in misidentification and mixed DNA profiles, which can possibly lead to a loss of crucial evidence and unsuccessful case solving. Therefore, effective anti-contamination measures in forensic laboratories must be applied. Commercial cleaning agents (ethanol and sodium hypochlorite) and ultraviolet C (UVC) radiation are commonly used for decontamination of working surfaces after casework analysis.

Moreover, this issue is especially important in accredited institutions like Forensic Science Centre “Ivan Vučetić” in Zagreb, Croatia. In Croatia, laboratories receive formal accreditation certificate from Croatian Accreditation Agency if they meet or exceed a list of standards according to HRN EN ISO/IEC 17025:2007. Accreditation certificate confirms competency, authority, and credibility of a forensic laboratory.

UV radiation is responsible for damage and mutations on DNA and tumor onset in humans (3). It is divided into UVA (wavelength 320-420 nm), UVB (wavelength 280-320 nm), and UVC (wavelength 200-280 nm) radiation (4,5). Ozone, oxygen, and vaporized water retain most of UVB radiation and all UVC radiation in the atmosphere. Nevertheless, DNA molecules absorb UVB and UVC photons, which could lead to accumulation of DNA damage and cause mutations. Most common forms of DNA damage induced by UV radiation are cyclobutane pyrimidine dimers, pyrimidine-pyrimidone UV photoproducts, and single and double-stranded DNA breaks (6). In living organisms, there are several repair mechanisms like photoreactivation, mismatch repair, nucleotide and base excision repair, recombination repair, and SOS response (7). Their goal is to preserve the integrity of DNA and prevent mutations. DNA isolated from biological evidence found at crime scenes is not under homeostatic control and can accumulate mutations with time, which could cause allele drop-outs in DNA profiles (8).

Recently, Hall and Ballantyne (8) have shown a complete loss of DNA profile after exposure of 50 μL dried blood trace on a filter paper to a UVC dose of 636 500 mJ/cm^2^. It remains to be answered if the resistance of blood to UVC radiation is a consequence of DNA conformation, along with the protective role of the cell, proteins, and RNA molecules, which absorb UVC radiation. DNA in solutions assumes standard B conformation which can form photoproducts after UVC photons absorption. On the other hand, dehydrated DNA assumes A conformation, which is not susceptible to formation of these structures. Similar research has been performed on an isolated DNA solution and dried DNA sample with the same DNA concentration values (8). UVC radiation dose needed for DNA profile loss was 90 times higher in the case of dried DNA sample than in the case of DNA solution (8).

Gefrides et al (9) investigated the influence of UVC radiation on saliva. They exposed 10 μL of dried saliva in the micro tube to UVC radiation for 180 minutes and detected 33% of alleles on genetic loci amplified with AmpFlSTR® Profiler Plus® (10) and AmpFlSTR® COfiler® kits (11) (DNA concentration 0.2 ng/μL; UVC dose 5616 mJ/cm^2^).

To our knowledge, the rate of UVC-induced DNA damage of semen stains has not been analyzed so far. DNA damage caused by the exposure of biological evidence to UVC radiation partially or entirely disables forensic DNA analysis by influencing polymerase chain reaction (PCR) efficiency and further DNA profile identification. The effect of UVC radiation on DNA persistence was analyzed in a few studies, mostly concentrating on sterilization of consumables in a crosslinker.

The aim of this study was to find favorable conditions for preventing DNA cross-contamination on working surfaces in laboratories by examining the influence of UVC radiation on naked DNA, diluted and undiluted blood, saliva, and semen samples in different time intervals. We also investigated the necessary doses of UVC radiation that would cause a complete loss of DNA profile in biological samples due to DNA degradation.

## Materials and methods

Reference buccal swab samples, blood, saliva, and semen were obtained from a single male donor, age 24 after informed consent had been obtained.

### Sample preparation

Four types of samples were used in this study: naked DNA (purified DNA sequence with no associated proteins) and diluted and undiluted blood, saliva, and semen. DNA was isolated from a buccal swab of the donor. One nanogram of DNA was dissolved in 10 μL of distilled water (DNA concentration 0.1 ng/μL). Three dilutions – 1:10, 1:20, and 1:50 were prepared from the initial concentration of 0.1 ng/μL. Blood, saliva, and semen were used undiluted (not in contact with water whatsoever) or they were diluted in the same way as naked DNA (the first dilution was made to achieve the same DNA concentration of 0.1 ng/μL, and from it 1:10, 1:20, and 1:50 dilutions were made).

Before starting the experiment, the working surface was cleaned with commercial cleaning agents (10% sodium hypochlorite and 20% ethanol, Kemika, Zagreb, Croatia) and 1.5 × 1.5 cm squares were drawn with a pencil. 10, 5, 3, and 1 μL of diluted and undiluted samples of naked DNA, blood, saliva, and semen, respectively were inflicted and smeared with a micropipette tip in triplicate on the prepared squares, and dried overnight.

### D+UVC and only-UVC test groups

After drying overnight, the working surface with naked DNA and diluted and undiluted samples was either decontaminated with 10% sodium hypochlorite (Kemika) and 20% ethanol (Kemika) and exposed to UVC radiation (test group D+UVC) or exposed to UVC radiation without decontamination (test group only-UVC).

### UVC exposure

The source of UVC radiation was Philips TUV 36W/G36 T8 in the laboratory and Philips TUV 15W/G15 T8 (Philips, Amsterdam, Netherlands) lamps in the laminar. The lamp was placed directly 193 cm above the working surface in the laboratory and 20 cm in the laminar. The same type of working surface was used in both experiments. During UVC exposure, the intensity of UVC radiation was measured with Radiometer Series 9811 (Cole-Parmer Instrument Company, Vernon Hills, IL, USA). The initial flux was 4.56 mJ/cm^2^ min in the laboratory and 153 mJ/cm^2^ min in the laminar. Exposure times for the experiments performed in the laminar were normalized to the initial flux rate and indicated as equivalents of the exposure times in the laboratory. After UVC exposure, D+UVC and only-UVC samples were swabbed with a cotton swab moistened with distilled water. The cotton parts of the swabs were cut with scissors and placed in separate micro tubes. Micro tubes containing negative controls (moistened cotton) were left open on the working surface during the whole experiment and closed after each time interval.

### Application of UVC radiation on D+UVC and only-UVC samples of the naked DNA and undiluted and diluted blood, saliva, and semen in experiments performed in the laboratory and laminar

Due to the different nature of samples, we used different volumes and time intervals for UVC exposure of D+UVC and only-UVC samples (Table 1).

**Table 1:** Experimental design*

Groups	Sample type	Initial DNA concentration (ng/µL)	Dilutions of initial DNA concentration	Volume (µL)	Location	Irradiation time (min, h)	UVC dose (mJ/cm^2^)
D+UVC only-UVC	Naked DNA	0.1	1:10, 1:20, 1:50	10	laboratory	2,5,15,30,45 min; 1,4,8,16 h	9-4380
	Diluted blood	0.1	1:10, 1:20, 1:50	10	laboratory	1 h	275
	Diluted saliva	0.1	1:10, 1:20, 1:50	10	laboratory	1 h	275
	Diluted semen	0.1	1:10, 1:20, 1:50	10	laboratory	1 h	275
	Undiluted blood	2.02	/	10	laboratory	1,4,8,16,24,32,42,54,66,78,90 h	275-24625
	Undiluted saliva	0.58	/	10	laboratory	1,4,8,16,24,32,42,54,66,78,90 h	275-24625
	Undiluted semen	20.55	/	10	laboratory	1,4,8,16,24,32,42,54,66,78,90 h	275-24625
only-UVC	Undiluted blood	2.02	/	10,5,3,1	laminar	Equivalent of: 42,66,90,120 180 250 h	11490-68400
	Undiluted semen	20.55	/	10,5,3,1	laminar	Equivalent of: 42,66,90,120 180 250 h	11490-68400

*Abbreviations: D+UVC – decontaminated samples exposed to UVC radiation; only-UVC – samples exposed to UVC radiation without decontamination.

### DNA analysis

Genomic DNA of D+UVC and only-UVC samples was extracted using Chelex (12). After isolation, the genomic DNA content of each sample was determined by quantitative real-time PCR using the Quantifiler^®^ Human DNA Quantification Kit (Applied Biosystems, Foster City, CA, USA) (13), which included an internal positive control to test for the presence of PCR inhibitors in isolated DNA. Quantitative real-time PCR was performed on a 7500 Real-Time PCR System (Applied Biosystems). Genomic DNA from D+UVC and only-UVC samples was amplified using the AmpFlSTR® NGM™ PCR Amplification Kit (Applied Biosystems) (14), which coamplifies 16 loci: D3S1358, vWA, D16S539, D2S1338, D8S1179, D21S11, D18S51, D19S433, TH01, FGA, D1S1656, D12S391, D10S1248, D22S1045, D2S441, and the sex marker Amelogenin. Genetic loci were divided into four groups depending on the median length of their alleles (Table 2). The amplification reactions were performed using a GeneAmp PCR System 9700 (Applied Biosystems). Amplification products were analyzed on 3130xl Genetic Analyzer (Applied Biosystems). Analysis of the data was performed using Genemapper^®^ software (version 3.2, Applied Biosystems). Amplicon sizing was conducted using an internal size standard (GeneScan-500 LIZ, Applied Biosystems), and the amplicons were compared with the AmpFlSTR^®^ NGM allelic ladder for unambiguous allele designation.

**Table 2:** Loci designation, number of alleles and repeats, and mean of allele length for each locus using AmpFlSTR® NGM™ PCR Amplification Kit

Loci designation	Number of alleles	Number of repeats	Mean of allele length (bp) ± standard deviation
**First group**			
D10S1248	11	8-18	76.64 ± 0.045 - 117.21 ± 0.044
D22S1045	12	8-19	79.99 ± 0.058 - 113.13 ± 0.059
D2S441	8	9-16	80.29 ± 0.054 - 104.53 ± 0.056
Amelogenin	2	/	100.54-100.68 ± 0.064; 106.2-106.34 ± 0.061
**Second group**			
D8S1179	12	8-19	123.54 ± 0.068 - 170.15 ± 0.096
D19S433	15	9-17.2	127.1 ± 0.05 - 161.66 ± 0.047
D3S1358	8	12-19	134.88 ± 0.064 - 164.06 ± 0.079
vWA	14	11-24	153.74 ± 0.065 - 206.12 ± 0.063
**Third group**			
D1S1656	16	9-20.3	175.77 ± 0.09 - 221.56 ± 0.064
TH01	10	4-13.3	181.53 ± 0.065 - 219.75 ± 0.047
D21S11	24	24-38	185.02 ± 0.069 - 240.71 ± 0.052
D16S539	9	5-15	228.68 ± 0.05 - 268.99 ± 0.054
**Fourth group**			
D12S391	15	14-27	229.97 ± 0.056 - 281.45 ± 0.066
FGA	28	17-51.2	233.22 ± 0.044 - 366.96 ± 0.052
D18S51	23	7-27	262.24 ± 0.068 - 344.5 ± 0.077
D2S1338	14	15-28	289.59 ± 0.054 - 343.05 ± 0.061

### Statistical analysis

Statistical data analysis was performed using Microsoft Excel 2010. The average DNA concentration (ng/µL), the number of correctly detected alleles on genetic loci amplified with AmpFlSTR® NGM™ PCR Amplification Kit (14), and the number of correctly detected alleles in four loci groups were tested, as well as the approximate UVC dose (mJ/cm^2^) values, sample volume (µL), and radiation time. Graphical data presentations were plotted using all data obtained in the research, not only average values for respective parameters.

## Results

### Determining the intensity of UVC radiation in the laboratory and laminar

The intensity of UVC radiation in the laboratory was 0.076 mW/cm^2^, and 2.55 mW/cm^2^ in the laminar. Flux values were calculated for the determination of the dose of UVC radiation at each time interval. Flux values were 4.56 mJ/cm^2^ min in the laboratory and 153 mJ/cm^2^ min in the laminar.

### Influence of UVC radiation performed in the laboratory on D+UVC and only-UVC samples of the naked DNA and undiluted and diluted blood, saliva, and semen

In D+UVC and only-UVC samples of the naked DNA, maximum DNA concentration collected after the treatment was 0.01 ng/μL. To determine the effects of UVC radiation on the ability to obtain a full genetic profile, UVC treated DNA samples were amplified using AmpFlSTR® NGM™ PCR Amplification Kit. A complete loss of DNA profile was detected in both D+UVC and only-UVC samples of the naked DNA already after 2 minutes.

Diluted blood, saliva, and semen (both D+UVC and only-UVC groups) were exposed to UVC radiation for 1 hour. Maximum DNA concentration obtained after UVC exposure in all tested samples was 0.01 ng/μL. After PCR amplification, a complete loss of DNA profile was observed in all cases.

In case of D+UVC samples of undiluted blood, a partial fourteen-locus DNA profile was detected even after 8 hours of UVC exposure. In longer time intervals (ie, 16 hours and longer), a complete loss of DNA profile was observed (Table 3). DNA concentration gradually decreased with higher UVC exposure time intervals. In case of only-UVC samples of undiluted blood, the full DNA profile was obtained even after 90 hours of UVC exposure in the laboratory, which was the longest exposure time tested.

**Table 3:** Initial volumes, time of exposure to UVC radiation, DNA concentrations, the number of correctly detected alleles on genetic loci, and approximate doses of UVC radiation for undiluted blood and semen in the laboratory*

Test group	Sample volume (µL)	Time of exposure to UVC radiation (h)	DNA concentration (ng/µL) (blood)	Number of detected alleles on genetic loci (blood)	DNA concentration (ng/µL) (semen)	Number of detected alleles on genetic loci (semen)	Dose of UVC radiation (mJ/cm^2^)
D+UVC	10	1	0.06	15/16	0.04	8/16	275
	10	4	0.04	14/16	0.03	6/16	1095
	10	8	0.03	14/16	-	-	2190
only-UVC	10	1	1.05	16/16	2.31	16/16	275
	10	4	0.67	16/16	3.96	16/16	1095
	10	8	0.76	16/16	3.19	16/16	2190
	10	16	0.49	16/16	0.96	16/16	4380
	10	24	0.24	16/16	2.3	16/16	6570
	10	32	0.2	16/16	1.77	16/16	8755
	10	42	0.13	16/16	1.73	16/16	11490
	10	54	0.09	16/16	0.84	15/16	14775
	10	66	0.07	16/16	1.61	16/16	18060
	10	78	0.04	16/16	1.29	15/16	21340
	10	90	0.05	16/16	1.21	14/16	24625

*Abbreviations: D+UVC – decontaminated samples exposed to UVC radiation; only-UVC ^-^– samples exposed to UVC radiation without decontamination.

In undiluted semen samples from D+UVC group, a partial eight-locus DNA profile was identified after 1 hour of UVC exposure. After 4 hours, a partial six-locus DNA profile was found (Table 3). In longer time intervals (ie, 8 hours and longer), none of the alleles on the analyzed genetic loci were detected. Quite opposite, a full DNA profiles were identified up to 42 hours of UVC exposure from samples of undiluted semen belonging to only-UVC group. Even after 90 hours of UVC radiation, a partial fourteen-locus DNA profile was observed (Table 3).

We also analyzed the influence of UVC radiation on undiluted saliva. In the samples belonging to D+UVC group and exposed for 1 hour to UVC radiation, a partial three-locus DNA profile was detected. In longer time intervals (ie, 4 hours and longer), a total DNA profile loss was observed. In only-UVC group, DNA concentrations were gradually decreasing with longer time intervals. Full DNA profiles were identified only after 1 hour of UVC exposure. After 42 hours of exposure, only partial two-locus DNA profile was identified (Table 4). After 54 hours of UVC exposure, a complete loss of DNA profile was detected.

**Table 4:** Initial volumes, time of exposure to UVC radiation, DNA concentrations, the number of correctly detected alleles on genetic loci, and approximate doses of UVC radiation for undiluted saliva samples from only-UVC group

Volume of saliva (µL)	Time of exposure to UVC radiation (h)	DNA concentration (ng/µL)	Number of correctly detected alleles on genetic loci	Dose of UVC radiation (mJ/cm^2^)
10	1	0.49	16/16	275
	8	0.15	9/16	2190
	16	0.11	6/16	4380
	42	0.07	2/16	11490

### Influence of UVC radiation on D+UVC and only-UVC samples of undiluted blood and semen in experiments performed in the laminar

**Figure 1:**
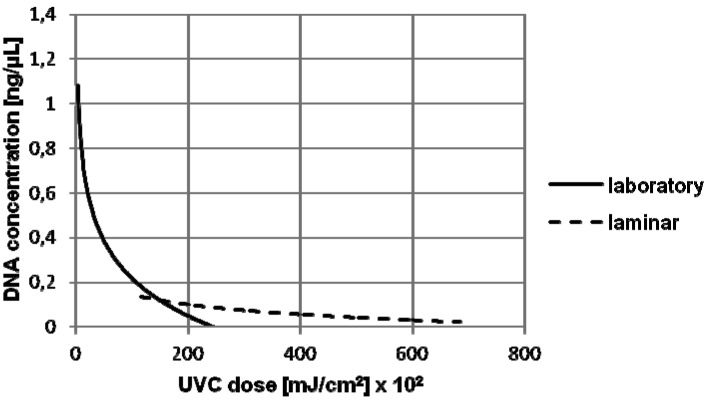
The correlation between DNA concentration and doses of UVC radiation for the experiments performed in the laboratory and laminar using 10 µL of undiluted blood treated only with UVC radiation (only-UVC group).

As even after 90 hours of UVC exposure of undiluted blood and semen belonging to the only-UVC group in the laboratory (Table 3), the goal of this study was still not achieved, the experiment was set up in the laminar, where 34 times higher UVC radiation was applied (Table 5). Figure 1 depicts the correlation between DNA concentration and doses of UVC radiation for the experiments performed in the laboratory and laminar using undiluted blood in the only-UVC group. Concentration values overlapped between the 11 490 and 24 625 mJ/cm^2^ UVC doses, which confirmed the correlation of results between the laboratory and laminar.

**Table 5:** Initial volumes, time of exposure to UVC radiation, DNA concentrations, the number of correctly detected alleles on genetic loci, and approximate doses of UVC radiation for undiluted blood and semen from only-UVC group in the laminar

Volume of blood or semen (µL)	Time of exposure to UVC radiation (h)	Dose of UVC radiation (mJ/cm^2^)	DNA concentration (ng/µL) (blood)	Number of correctly detected alleles on genetic loci (blood)	DNA concentration (ng/µL)(semen)	Number of correctly detected alleles on genetic loci (semen)
10	42	11490	0.28	16/16	1.52	15/16
	90	24625	0.09	16/16	1.2	13/16
	180	49250	0.06	16/16	0.92	9/16
	250	68400	0.03	16/16	0.47	6/16
5	42	11490	0.1	16/16	0.61	12/16
	90	24625	0.06	16/16	0.41	9/16
	180	49250	0.03	13/16	0.16	4/16
	250	68400	0.02	12/16	0.2	4/16
3	42	11490	0.09	10/16	0.23	8/16
	90	24625	0.03	1/16	0.24	6/16
	180	49250	0.03	1/16	0.09	3/16
	250	68400	0.01	0/16	0.1	3/16
1	42	11490	0.03	1/16	0.13	7/16
	90	24625	0.03	0/16	0.1	4/16
	180	49250	0.01	0/16	0.04	1/16
	250	68400	0.01	0/16	0.04	1/16

**Figure 2:**
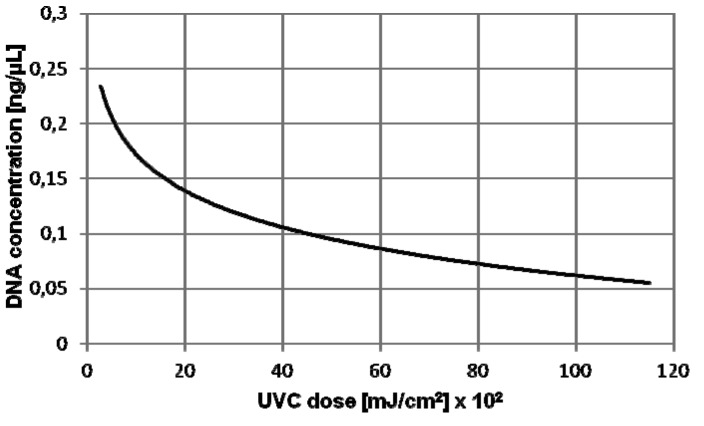
The correlation between DNA concentration and doses of UVC radiation for the experiments performed in the laboratory and laminar using 10 µL of undiluted saliva treated only with UVC radiation (only-UVC group).

**Figure 3:**
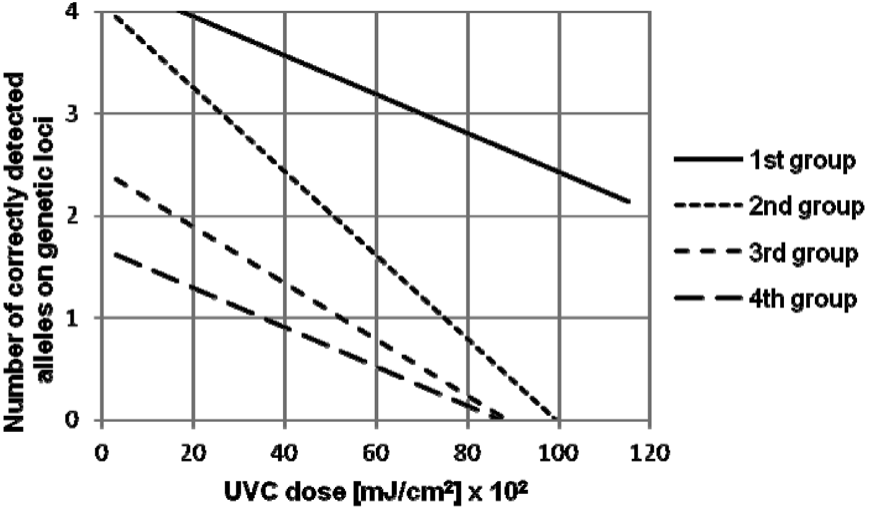
The correlation between the number of correctly detected alleles on analyzed genetic loci in four previously defined genetic loci groups and doses of UVC radiation, for the experiments performed in the laboratory using 10 μL of undiluted saliva treated only with UVC radiation (only-UVC group).

**Figure 4:**
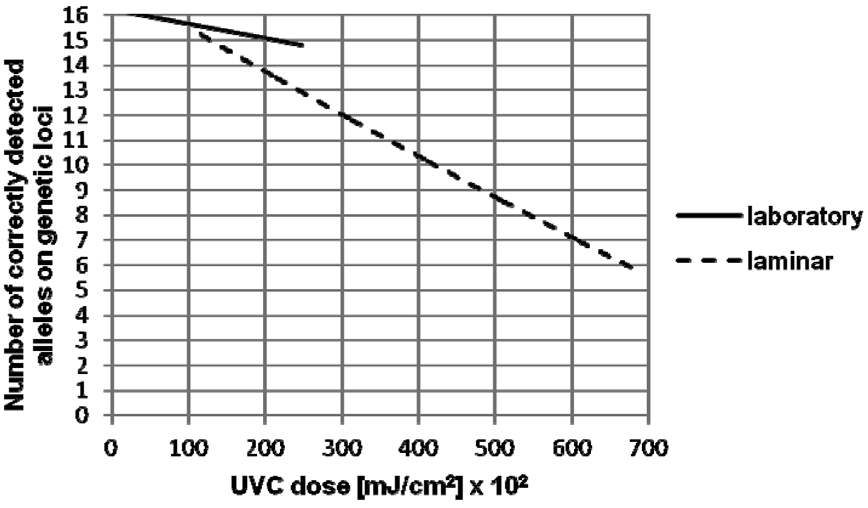
The correlation between the number of correctly detected alleles on genetic loci and doses of UVC radiation for the experiments performed in the laboratory and laminar using 10 µL of undiluted semen treated only with UVC radiation (only-UVC group).

**Figure 5:**
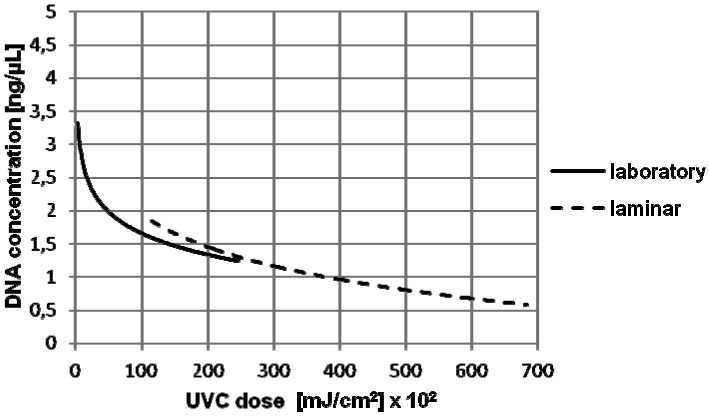
The correlation between DNA concentration values and doses of UVC radiation for the experiments performed in the laboratory and laminar using 10 µL of undiluted semen treated only with UVC radiation (only-UVC group).

**Figure 6:**
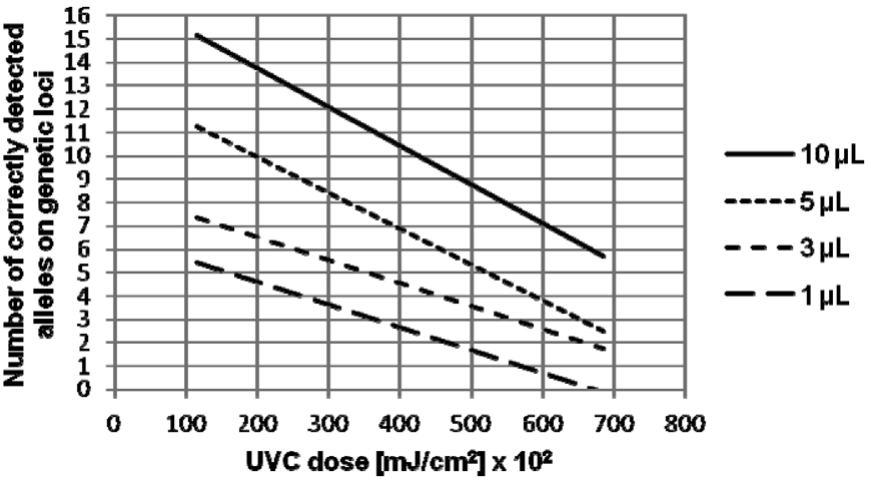
The correlation between the number of correctly detected alleles and doses of UVC radiation for the experiments performed in the laminar using 10, 5, 3, and 1 μL of undiluted semen treated only with UVC radiation (only-UVC group).

**Figure 7:**
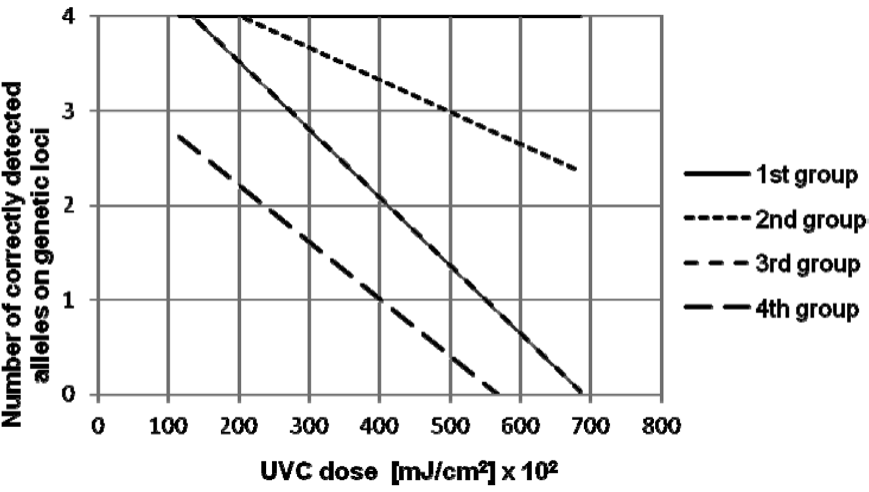
The correlation between the number of correctly detected alleles on analyzed genetic loci in four previously defined genetic loci groups and doses of UVC radiation, for the experiments performed in the laminar using 10 μL of undiluted semen treated only with UVC radiation (only-UVC group).

A decrease in the number of correctly detected alleles due to DNA degradation in undiluted saliva and semen from only-UVC group was obtained when higher UVC doses were applied (Figures 2-7). Still, in the case of semen, even with the longest UVC exposure tested (equivalent of 250 hours, ie, more than 10 days) in the laboratory, the complete loss of genetic profile was not achieved. The same occurred with higher amounts of blood samples, while the complete loss of genetic profile was achieved with smaller amounts of blood, but 90-hour (ie, almost 4 days) or longer UVC exposure was needed.

## Discussion

We showed that DNA profiles from naked DNA were not identified after 2 minutes of UVC exposure in the laboratory, due to rapid DNA degradation. Hall and Ballantyne (8) exposed 100 ng/μL of dried DNA to UVC dose of 149750 mJ/cm^2^ in a crosslinker, which caused a complete DNA profile loss. In our study, the initial concentration was much lower (0.1 ng/µL), as well as the amount of radiation, therefore our results are complementary with those of Hall and Ballantyne. As the samples in the water are also very sensitive, the genetic profile could not be generated in diluted blood, saliva, and semen after 1 hour of UVC exposure.

In the case of saliva, Gefrides et al (9) exposed 10 μL of dried saliva in the micro tube to UVC radiation for 180 minutes. They detected 33% of alleles on genetic loci amplified with AmpFlSTR® Profiler Plus® (10) and AmpFlSTR® COfiler® PCR Amplification kits (11) (DNA concentration 0.2 ng/μL; UVC dose 5616 mJ/cm^2^), while 100% of the alleles were detected using AmpFlSTR® Minifiler^TM^ PCR Amplification Kit (15). In our case, when only-UVC radiation was used, a partial genetic profile was obtained even after 42 hours of radiation. Still if the working surface was cleaned before UVC radiation, 4 hours were enough for a complete loss of DNA profile.

In the case of undiluted blood, in our study a full DNA profile was detected (DNA concentration after radiation 0.06 ng/μL; UVC dose 32835 mJ/cm^2^) when 10 μL of samples were exposed in the laminar to the equivalent of 120 hours of UVC radiation, in the group where no decontamination was performed. Quite contrary, in a similar study, Hall and Ballantyne (8) exposed 50 μL of dried blood on filter paper in a crosslinker for 120 hours to a UVC dose of 636.5 J/cm^2^ and a complete loss of DNA profile was observed. Still, the results of these two studies cannot be compared due to a different experimental setup.

This is the first study dealing with the effects of UVC radiation on semen. Undiluted semen showed slower and irregular lowering of DNA concentration than blood. The reason for this may be a significantly higher initial DNA concentration or the nature of semen. As it was mentioned before, blood is resistant to UVC radiation due to the protective role of the cell as a whole and RNA molecules and proteins, which absorb the radiation. Semen is most probably resistant to UVC radiation due to the same reasons.

When analyzing the results of this research, one must bear in mind that several parameters could influence DNA concentration after the treatment, such as variations in UVC dose caused by samples positioning on the working surface, distance between the working surface and UVC source, incomplete swabbing of samples, use of a cotton swab that was insufficiently or overly moist for samples swabbing and DNA isolation, as well as uneven pipetting during PCR and RT-PCR preparation.

Longer alleles in undiluted saliva and semen dropped out before shorter ones when exposed to UVC radiation for longer periods of time. According to the study by Champlot et al (16), these results were expected. The authors examined the influence of UVC radiation on DNA fragments of various lengths and concluded that longer fragments exhibited higher susceptibility to DNA degradation than shorter ones. Also, they pointed out that higher UVC dose was necessary for the same degradation level and that the influence of UVC radiation on DNA degradation was reversely proportional to the distance of UVC radiation source.

It is interesting to compare the UVC doses used in this research with the time equivalent of biological sample exposure to midday sun. A UVC dose of 42 mJ/cm^2^ is equivalent to 20 hours of biological sample exposure to midday sun (8). Knowing this fact, it is easy to calculate that after 2085 hours (87 days) of exposure to midday sun, partial DNA profile could not be identified from 10 μL of undiluted saliva. The DNA profile of the donor also could not be identified after 32 570 hours of exposure of 10 μL of undiluted semen, as well as after 8600 hours of exposure of 3 μL of undiluted blood. Other environmental factors also have to be taken into account. Moisture, type of surface on which the sample was exposed, and temperature variations could also cause DNA degradation, reducing the time period when partial DNA profile identification is possible.

Contamination may occur in every laboratory and even the most stringent anti-contamination protocols fail. One of the examples is ancient DNA laboratory working with skeletal remains, where contamination with exogenous DNA may occur. The highest level of anti-contamination measures should be implemented in these laboratories to prevent identification of mixed or inauthentic DNA profiles (17).

Laboratory accreditation is the most important step in assessing the quality of a forensic laboratory. Various anti-contamination measures must be implemented to assure such high quality standards. Anti-contamination measures include protective clothes (mask, cap, laboratory coat, and gloves), spatial and temporal separation of casework analysis, and sterilization of all laboratory equipment before use by UVC radiation to avoid contamination with foreign DNA, and ordering laboratory equipment from verified sources. Also, the usage of positive and negative controls and blanks are obligatory, because they serve as a contamination indicator, by giving important information about the functionality of reagents and equipment during analysis.

In conclusion, high UVC doses with prior cleaning of the working surface (D+UVC group) were necessary for complete decontamination of working surfaces. For this reasons, it is recommended to carefully and thoroughly clean working surfaces with 10% sodium hypochlorite and 20% ethanol followed by minimal 16-hour UVC exposure (dose approximately 4380 mJ/cm^2^) for complete and successful decontamination.

##
